# Seroprevalence of Cutaneous Human Papillomaviruses and the Risk of External Genital Lesions in Men: A Nested Case-Control Study

**DOI:** 10.1371/journal.pone.0167174

**Published:** 2016-11-28

**Authors:** Shams Rahman, Dana E. Rollison, Christine M. Pierce Campbell, Tim Waterboer, Angelika Michel, Michael Pawlita, Luisa L. Villa, Eduardo Lazcano Ponce, Wei Wang, Amy R. Borenstein, Anna R. Giuliano

**Affiliations:** 1 Center for Infection Research in Cancer, Moffitt Cancer Center, Tampa, United States; 2 Department of Epidemiology and Biostatistics, College of Public Health, University of South Florida, Tampa, United States; 3 Infection, Inflammation and Cancer Research Program, German Cancer Research Center (DKFZ), Heidelberg, Germany; 4 School of Medicine, University of São Paulo, São Paulo, Brazil; 5 Instituto Nacional de Salud Publica, Cuernavaca, Mexico; Baylor College of Medicine, UNITED STATES

## Abstract

**Background:**

A variety of cutaneous human papillomaviruses (HPV) are detectable in genital epithelial lesions in men and non-melanoma skin cancer patients. It remains unclear whether these viruses are associated causally with skin lesions. To date, no study has prospectively examined the association between cutaneous HPV seropositivity and development of external genital lesions (EGLs) in men.

**Objectives:**

To examine the association between seropositivity to cutaneous HPV types and the risk of subsequent development of EGLs.

**Methods:**

A nested case-control study including 163 incident EGL cases and 352 EGL-free controls in *the HPV Infection in Men (HIM) Study* cohort was conducted. Cases were ascertained at each of up to 10 biannual clinical visits and verified through biopsy and pathological diagnoses. EGLs were categorized as condyloma, suggestive of condyloma, penile intraepithelial neoplasia (PeIN), and other EGLs. Archived serum specimens collected at baseline were tested for antibodies against 14 cutaneous HPV types (β types (5, 8, 12, 14, 17, 22, 23, 24, 38, and 47), α type 27, γ type 4, μ type 1, and ν type 41) using a GST L1-based multiplex serology assay. Socio-demographic and sexual behavior data were collected through a questionnaire. Using logistic regression, adjusted odds ratios (AOR) and 95% confidence intervals (CI) were estimated.

**Results:**

Overall, seropositivity to ≥1 cutaneous HPV type (any-HPV) and ≥1 β types (any-β) was 58.3% and 37.5% among other EGL cases, 71.6% and 46.8% among condyloma, 66.8% and 50.0% among PeIN, and 71.9% and 38.4% among controls, respectively. Type-specific seropositivity was most common for ɤ-HPV 4, μ-HPV 1, and β-HPV 8. No statistically significant association was observed between any-HPV, any-β, and type-specific HPV seropositivity and subsequent development of EGLs across all pathological diagnoses.

**Conclusions:**

Overall, seropositivity to cutaneous HPV was common among men; however, it appears that cutaneous HPV is not associated with the development of genital lesions in men.

## Introduction

Over 200 types of human papillomaviruses (HPV) have been identified,[1] and classified into five genera: alpha (α), beta (β), gamma (γ) mu (μ) and nu (ν) [2, 3]. The majority of HPV in the genus α infect mucosal membranes but some types are also detected in cutaneous skin [4, 5]. HPV with cutaneous tropism are categorized into genus β, including some types initially referred to as epidermodysplasia verruciformis (EV) types, which have been associated with skin lesions, particularly in immune-suppressed individuals. Genus γ infects cutaneous skin and creates intra-cytoplasmic inclusion bodies. Finally, genera μ and ν are also associated with skin lesions [4, 5].

Emerging evidence shows that cutaneous HPV infection may increase the risk of squamous cell carcinoma (SCC) of the skin [6, 7]. Several studies have reported a positive association between cutaneous HPV seropositivity and DNA detection and the risk of SCC [5, 8–11]. It is hypothesized that cutaneous HPV infection might modify the effect of ultraviolet (UV) radiation induced DNA damage and apoptosis leading to accumulation of mutations and SCC [12]. Cutaneous HPV DNA has also been detected on the surface of penile intraepithelial neoplasia (PeIN) [13, 14], penile cancer [15, 16], genital warts and other EGLs [17] and skin warts [18]. Some EGLs such as PeIN are precursors of penile cancer [19]. Less prevalent in the United States[20], penile carcinoma constitutes up to 10% of all cancers among men in low-resource countries[21], and its incidence is on the rise in some European countries[22–24]. Genital warts (condyloma acuminata) are a common sexually transmitted infection (STI). Although benign lesions, genital warts cause considerable amount of psychological discomfort and treatment related burden to patients [25]. Both genital warts and PeIN (low-grade and high-grade) are associated with an increased risk for carcinoma of the penis [26, 27].

The detection of cutaneous HPV DNA on the surface of EGLs may suggest a role in the pathogenesis of the squamous epithelium lesions in the UV unexposed areas of the body. However, little data exist on the epidemiology and serology of cutaneous HPV and their distributions and etiologic role in the development of EGLs. Previously we detected β-HPV DNA on the surface of 61.1% of all EGLs [17]. Subsequently, in a nested case-control study *(Campbell et al*. *manuscript under review)* we detected a lower prevalence of β-HPV DNA on the surface of EGLs compared to controls, and found that some β-HPV types were inversely associated with condyloma. To our knowledge, no study has yet investigated the association of seropositivity to cutaneous HPV and the risk of EGLs among men prospectively. The purpose of this study was to examine the association between seropositivity to cutaneous HPV types and the risk of subsequent development of EGLs among men in a case-control study nested in the *HIM* cohort.

## Material and Methods

### Study Population

This nested case-control study evaluated 163 incident EGL cases and 352 EGL-free controls in the *HIM* cohort, a multinational prospective study of the natural history of HPV infection in men. Study population and methods have been described previously in details [28, 29]. Briefly, between July 2005 and September 2009, the *HIM Study* enrolled over 4000 men in the United States, Brazil, and Mexico. Participants were eligible if they were: male, aged 18–70 years at baseline, reported no previous diagnosis of penile or anal cancer, had no previous diagnoses of ano-genital warts, no current history or treatment for sexually transmitted infections including HIV, and no current discharges from the penis or burning sensation during urination. Participants were followed every six months for a median of four years. At each study visit, participants completed a computer-assisted self-interviewed questionnaire provided urine and blood samples, and underwent a clinical examination.

Cases included pathologically confirmed incident EGLs ascertained through visual inspection and confirmed with pathology following biopsy. The *HIM Study* pathology protocol was implemented in February 2009. Approximately 2,754 men, who had ≥2 study visits approximately six months apart were included in the biopsy sub-cohort, described previously in details [30, 31]. Briefly, at each visit, a trained clinician examined men under 3x light magnification for EGLs. Using shave excision, a tissue specimen was collected from each lesion. Excised tissues were placed in 10% buffered formalin, sent to the University of South Florida Dermatopathology Laboratory and evaluated by two independent pathologists. Discrepant diagnoses were adjudicated by a panel of three independent pathologists and quality control was conducted on 10% of all biopsy specimens [30]. Using classification criteria published before [30, 32], EGLs were categorized into four groups: i) condyloma, ii) suggestive of condyloma, iii) PeIN, and iv) other EGL cases. A lesion with koilocytes, papillomatosis, hypergranulosis, parakeratosis and dilated blood vessels was diagnosed as ‘condyloma’, and a lesion without koilocytes but with one or two of the other features associated with a condyloma was considered ‘suggestive of condyloma’. These lesions were most likely early condyloma that did not manifest complete histological features of a fully developed condyloma. ‘Condyloma’ and ‘suggestive of condyloma’ were therefore combined in the statistical analyses (i.e. the combined condyloma group). Lesions such as molluscum contagiosum, intradermal nevus, fibroepithelial polyp (skin tag), chronic balanitis, genital melanotic macule, psoriasiform dermatitis, lichenoid tissue reaction, and acute mucositis, which had unknown etiology on gross examination, but presented definite histology on pathologic review, were categorized into the ‘other EGL cases’ group. Finally, lesions with pre-neoplastic or neoplastic cells were categorized as ‘PeIN’. Additionally, all four categories of pathological diagnoses were combined into one group ‘all EGL cases’ in the multivariable models.

Controls included *HIM Study* participants who did not develop an EGL throughout the entire four years of follow-up. Two controls, frequency matched on the length of follow up, were selected per case. All participants provided written informed consent. All study procedures were approved by the Institutional Review Boards of the University of South Florida (Tampa, FL, USA), the Ludwig Institute for Cancer Research (Sao Paulo, Brazil), the Centro de Referencia e Treinamento em Doencas Sexualmente Transmissiveis e AIDS (Sao Paulo, Brazil), and the Instituto Nacional de Salud Publica de Mexico (Cuernavaca, Mexico).

### Specimen and Data Collection

At each study visit, *HIM Study* participants provided detailed information on demographics (age, race, ethnicity, education and marital status), socioeconomic status, medical history, smoking habits, alcohol consumption, and sexual history. Archived biopsy cohort baseline serum specimens among both cases and controls were tested for seroreactivity to the L1 protein of 14 cutaneous HPV types including β types (5, 8, 12, 14, 17, 22, 23, 24, 38, and 47), α type 27, γ type 4, μ type 1, and ν type 41. Only 14 types were tested due to limited funds, and were carefully selected based on previous reports of their association with cutaneous SCC [8, 11], and their detection on the surface of EGLs [17]. The antibody detection method was based on a glutathione S-transferase (GST) capture enzyme-linked immunosorbent assay (ELISA), in combination with fluorescent bead technology (Luminex) as previously described [33–35]. To define type-specific HPV seropositivity, standard cut-off values (200 median fluorescence intensity [MFI]) were applied, as previously described [36, 37].

### Statistical Analysis

Demographic, lifestyle and sexual behavior characteristics were compared between cases and controls using Chi-square or Fisher’s exact test (when ≥1 cells had an expected frequency of ≤5) for categorical variables (Table 1). Type-specific seroprevalence was defined as the proportion of men who tested seropositive for antibodies to a given HPV type. Seroprevalence to any cutaneous HPV (any-HPV) was defined as the proportion of men who were seropositive to at least one of the 14 types of HPV included in this study, and seroprevalence to any-β HPV was defined as the proportion of men who were seropositive to at least one of the 10 β-HPV types. Type-specific and grouped seroprevalence was calculated for different diagnostic categories of EGLs and controls. Seropositivity to one type, two types, and ≥3 types was also estimated. To assess for confounding, associations between factors listed in Table 1, and any-HPV, any-β, and type-specific HPV seropositivity were examined among controls; associations between these factors and EGL development were assessed among the seronegative groups (unexposed) for any-HPV, any-β, and type-specific HPV. Also, factors associated with the cutaneous HPV seroprevalence, reported previously for the same population (*manuscript under review*), were also considered for inclusion in the multivariable modeling. The final multivariable model included country, age, education, circumcision status, lifetime number of female sex partners, and lifetime number of male anal sex partners. Using these adjustment factors and logistic regression, separate models were estimated for associations between seropositivity to any-HPV type, any-β HPV type, and each specific HPV type, and EGL development. Interaction with seropositivity to nine mucosal HPV vaccine types (6, 11, 16, 18, 31, 33, 45, 52 and 58) was also assessed. Additionally, it was hypothesized that cutaneous HPV seropositivity could be associated with condyloma with definite HPV 6/11 etiology. Therefore, stratified associations were evaluated in the condyloma group by tissue DNA positivity to HPV 6/11 and antibody status to HPV 6/11. All analyses were performed in SAS 9.3.

**Table 1:** Association between demographic, lifestyle and sexual behavior factors and external genital lesions (EGLs) and controls.

Characteristics	EGLs Other than Conlyloma, Suggestive of Condyloma and PeIN^a^ (N = 48)			Condyloma^b^ and Suggestive of Condyloma^c^ (N = 109)			PeIN^d^ (N = 6)			Control (N = 352)	
	n	(%)	p^e^	n	%	p^e^	n	%	p^e^	n	%
**Country**											
USA	1	2.1	**<0.001**	32	29.4	0.193	1	16.7	0.275	137	38.9
Brazil	19	39.6		45	41.3		4	66.7		124	35.2
Mexico	28	58.3		32	29.4		1	16.7		91	25.9
**Age, Years**											
18–30	13	27.1	0.264	50	45.9	0.235	3	50.0	0.496	135	38.4
31–44	26	54.2		45	41.3		3	50.0		151	42.9
45–73	9	18.8		14	12.8		0	0.0		66	18.8
**Race**											
White	11	22.9	**<0.001**	60	55.0	0.160	4	66.7	0.135	182	51.7
Black	9	18.8		15	13.8		1	16.7		65	18.5
Asian	0	0.0		1	0.9		0	0.0		15	4.3
Native Hawaiian/Pacific Islanders	0	0.0		1	0.9		0	0.0		0	0.0
American Indian, Alaska Native	1	2.1		2	1.8		1	16.7		3	0.9
Others	27	56.3		29	26.6		0	0.0		79	22.4
Not reported	0	0.0		1	0.9		0	0.0		8	2.3
**Ethnicity**											
Hispanic	34	70.8	**0.002**	46	42.2	0.922	1	16.7	0.478	142	40.3
Non-Hispanic	13	27.1		63	57.8		5	83.3		207	58.8
Not reported	1	2.1		0	0.0		0	0.0		3	0.9
**Education, Years**											
12 or less	27	56.3	**0.028**	49	45.0	0.416	4	66.7	0.681	148	42.0
13–15	6	12.5		27	24.8		1	16.7		99	28.1
16 or more	12	25.0		33	30.3		1	16.7		98	27.8
Not reported	3	6.3		0	0.0		0	0.0		7	2.0
**Marital Status**											
Single/never married	9	18.8	**0.003**	53	48.6	0.076	2	33.3	0.264	139	39.5
Married	23	47.9		28	25.7		2	33.3		132	37.5
Cohabitating, living together	14	29.2		15	13.8		0	0.0		48	13.6
Divorced/separated/widowed	2	4.2		12	11.0		2	33.3		33	9.4
Not reported	0	0.0		1	0.9		0	0.0		0	0.0
**Smoking Status**											
Current	12	25.0	0.979	35	32.1	0.253	2	33.3	0.061	85	24.1
Former	7	14.6		14	12.8		3	50.0		49	13.9
Never	29	60.4		60	55.0		1	16.7		218	61.9
**Alcohol, No. of Drinks/Month**											
0	13	27.1	0.637	17	15.6	0.061	2	33.3	0.762	87	24.7
1–30	27	56.3		51	46.8		3	50.0		171	48.6
31–60	4	8.3		12	11.0		1	16.7		38	10.8
61 or more	4	8.3		29	26.6		0	0.0		54	15.3
Not reported	0	0.0		0	0.0		0	0.0		2	0.6
**Circumcision**											
No	40	83.3	**0.001**	69	63.3	0.347	5	83.3	0.407	205	58.2
Yes	8	16.7		40	36.7		1	16.7		147	41.8
**Sexual Orientation**											
MSW	40	83.3	0.489	94	86.2	0.385	6	100.0	0.470	310	88.1
MSM	5	10.4		10	9.2		0	0.0		29	8.2
MSWM	2	4.2		5	4.6		0	0.0		8	2.3
Missing	1	2.1		0	0.0		0	0.0		5	1.4
**Lifetime Number of Female Sex Partners**											
0	3	6.3	0.334	10	9.2	0.618	0	0.0	0.464	36	10.2
1–3	5	10.4		14	12.8		0	0.0		57	16.2
4–18	27	56.3		44	40.4		2	33.3		146	41.5
19 or more	11	22.9		35	32.1		4	66.7		103	29.3
Not reported	2	4.2		6	5.5		0	0.0		10	2.8
**Lifetime Number of Male Anal Sex Partners**											
0	41	85.4	0.726	91	83.5	0.162	6	100.0	0.764	295	83.8
1–3	1	2.1		3	2.8		0	0.0		11	3.1
4 or more	3	6.3		9	8.3		0	0.0		13	3.7
Not reported	3	6.3		6	5.5		0	0.0		33	9.4
**Recent Female Sex Partner, #**^f^											
0	6	12.5	0.696	14	12.8	**0.043**	0	0.0	0.443	46	14.6
1	25	52.1		39	35.9		3	50.0		170	53.8
2 or more	14	29.2		44	40.4		3	50.0		94	29.8
Not reported	3	6.3		12	11.0		0	0.0		6	1.9
**Recent Male Sex Partners, #** ^f^											
0	42	87.5	0.332	91	83.5	0.343	6	100.0	0.836	308	88.5
1	3	6.3		4	3.7		0	0.0		8	2.3
2 or more	1	2.1		6	5.5		0	0.0		9	2.6
Not reported	2	4.2		8	7.3		0	0.0		23	6.6

EGLs = external genital lesions; PeIN = penile intraepithelial neoplasia

^a.^ This category is also referred to as ‘other EGL cases’ which included different other diagnoses i.e. molluscum contagiosum, intradermal nevus, fibroepithelial polyp (skin tag), chronic balanitis, genital melanotic macule, psoriasiform dermatitis, lichenoid tissue reaction, and acute mucositis.

^b.^ Condyloma: A lesion with koilocytes, papillomatosis, hypergranulosis, parakeratosis and dilated blood vessels.

^c.^ Suggestive of condyloma: A lesion without koilocytes but with one or two of the other features associated with a condyloma. These lesions were most likely early condyloma that did not show complete histological features of a fully developed condyloma.

^d.^ PeIN: A lesion with pre-neoplastic or neoplastic cells.

^e.^ P-values (p) compares respective EGL category with the last column (control group). P-values were calculated using Chi-square or Fisher’s exact tests (when ≥1 cells had an expected frequency of ≤5), significant p-values are highlighted in bold.

^f.^ Number of female sex partners in the past 6 months among those reporting ever having a female sex partner; number of male sex partners in the past 6 months among those reporting ever having a male sex partner.

## Results

The characteristics of the 163 incident EGL cases and 352 controls are shown in Table 1. After a median follow up of 19 months (IQR 8–48 months) among 48 men diagnosed with other EGL cases, statistically significant differences (p<0.05) by country, race, ethnicity, education, marital status, and circumcision were observed when compared to controls. Men from Brazil and Mexico, the “other” race group, Hispanics, men with ≤12 years of education, and uncircumcised men were more likely to have other EGLs diagnoses compared to controls. In total, 109 men were diagnosed with condyloma (n = 62) or suggestive of condyloma (n = 47). Significant differences by the number of recent female sex partners among those reporting ever having a female sex partner were observed for the combined group of condyloma. Men with female sex partners were more likely to develop condyloma. Also, men with female partner (life time or recent) were more likely to develop EGLs than men with male partners (life time or recent). Six men were diagnosed with PeIN. Significant differences between PeIN and controls were not observed for any of the socio-demographic and behavioral characteristics evaluated.

Table 2 presents seropositivity of type-specific and grouped cutaneous HPV by case-control status. Approximately, 58.3% and 37.5% of men in the other EGL cases group, 71.6% and 46.8% in the combined condyloma, 66.8% and 50.0% in the PeIN, and 71.9% and 38.4% in the control group were seropositive for any-HPV and any-β HPV, respectively. The most commonly occurring type-specific HPV were ɤ-HPV 4, μ-HPV 1, and β-HPV 8. Approximately 25.0% of men in the other EGL cases group, 31.2% in the combined condyloma, 50.0% in the PeIN, and 31.0% in the control group were seropositive for ɤ-HPV 4. Approximately 14.6% and 22.9% of men in the other EGL cases group, 25.7% and 22.9% in the combined condyloma, and 33.3% and 33.3% in the PeIN, and 31.3% and 19.6% in the control group were seropositive for μ-HPV 1 and β-HPV 8, respectively. Seropositivity for only one HPV type ranged from (16.7%) to (35.5%), for two HPV types from (10.4%) to (20.2%), and for ≥3 HPV types from (22.0%) to (33.3%) across different pathologic diagnoses.

**Table 2:** Grouped and type-specific cutaneous HPV seropositivity by case-control status.

HPV type	EGLs^a^ Other than Condyloma, Suggestive of Condyloma and PeIN (N = 48)		Condyloma and Suggestive of Condyloma (N = 109)^b^^,^^c^		PeIN^d^(N = 6)		Controls(N = 352)	
	n	%	n	%	n	%	n	%
**Any-HPV**								
Seropositive	28	58.3	78	71.6	4	66.8	253	71.9
**Any β HPV**								
Seropositive	18	37.5	51	46.8	3	50.0	135	38.4
**α-HPV 27**								
Seropositive	8	16.7	13	11.9	0	0.0	34	9.7
**γ-HPV 4**								
Seropositive	12	25.0	34	31.2	3	50.0	109	31.0
**μ-HPV 1**								
Seropositive	7	14.6	28	25.7	2	33.3	110	31.3
**ν-HPV 41**								
Seropositive	4	8.3	10	9.2	1	16.7	42	11.9
**β-HPV 5**								
Seropositive	3	6.3	9	8.3	1	16.7	32	9.1
**β-HPV 8**								
Seropositive	11	22.9	25	22.9	2	33.3	69	19.6
**β-HPV 12**								
Seropositive	4	8.3	5	4.6	1	16.7	20	5.7
**β-HPV 14**								
Seropositive	3	6.3	2	1.8	1	16.7	17	4.8
**β-HPV 17**								
Seropositive	6	12.5	20	18.4	2	33.3	55	15.6
**β-HPV 22**								
Seropositive	3	6.3	9	8.3	1	16.7	21	6.0
**β-HPV 23**								
Seropositive	4	8.3	15	13.8	0	0.0	36	10.2
**β-HPV 24**								
Seropositive	1	2.1	5	4.6	1	16.7	17	4.8
**β-HPV 38**								
Seropositive	8	16.7	14	12.8	0	0.0	46	13.1
**β-HPV 47**								
Seropositive	4	8.3	13	11.9	1	16.7	51	14.5
**Seropositivity to 1, 2, 3 or more HPV types**								
Seropositive to 1 HPV	12	25.0	32	29.4	1	16.7	125	35.5
Seropositive to 2 HPV	5	10.4	22	20.2	1	16.7	57	16.2
Seropositive to 3 or more HPV	11	22.9	24	22.0	2	33.3	71	20.2

EGLs = external genital lesions; PeIN = penile intraepithelial neoplasia

^a.^ This category is also referred to as ‘other EGL cases which included different other diagnoses i.e. molluscum contagiosum, intradermal nevus, fibroepithelial polyp (skin tag), chronic balanitis, genital melanotic macule, psoriasiform dermatitis, lichenoid tissue reaction, and acute mucositis.

^b.^ Condyloma: A lesion with koilocytes, papillomatosis, hypergranulosis, parakeratosis and dilated blood vessels.

^c.^ Suggestive of condyloma: A lesion without koilocytes but with one or two of the other features associated with a condyloma. These lesions were most likely early condyloma that did not show complete histological features of a fully developed condyloma.

^d.^ PeIN: A lesion with pre-neoplastic or neoplastic cells.

The univariate and multivariable associations of grouped and type-specific cutaneous HPV seropositivity and each of the three case outcomes (all EGL cases, combined condyloma, and other EGL cases) are presented in Table 3. No association was observed between any-HPV, any-β, and type-specific HPV seropositivity and ‘all EGL’ cases in either the univariate or the multivariable analyses, except for μ-HPV 1. Seropositivity to μ-HPV 1 was significantly inversely associated with ‘other EGL cases’ and ‘all EGL cases’ categories in the unadjusted models. However, after controlling for confounding, this significance was lost (adjusted odds ratio ([AOR] 0.62; 95% confidence interval [CI:] 0.31–1.65) for ‘other EGL cases’ and (AOR 0.78; 95%CI: 0.48–1.28) for ‘all EGL cases’ category), respectively. Similarly, no association was observed between any-HPV, any-β, and type-specific HPV seropositivity and ‘combined condyloma’ and ‘other EGL’ cases in either the univariate or the multivariable analyses, except for μ-HPV 1.

**Table 3:** Association between grouped and type-specific cutaneous HPV seropositivity and EGLs^a^.

HPV type	EGLs Other than Condyloma Suggestive of Condyloma and PeIN^b^		Condyloma and Suggestive of Condyloma^c^		All EGL Cases^d^	
	OR	AOR^e^ 95% CI	OR	AOR^e^ 95% CI	OR	AOR^e^ 95% CI
**Any-HPV**						
Negative	1.00	1.00	1.00	1.00	1.00	1.00
Positive	0.55	0.63 (0.30–1.32)	0.98	1.03 (0.61–1.72)	0.81	0.91 (0.58–1.42)
**Any β HPV**						
Negative	1.00	1.00	1.00	1.00	1.00	1.00
Positive	0.96	1.23 (0.59–2.56)	1.41	1.53 (0.95–2.47)	1.27	1.46 (0.95–2.24)
**α-HPV 27**						
Negative	1.00	1.00	1.00	1.00	1.00	1.00
Positive	1.87	2.31 (0.88–6.05)	1.27	1.24 (0.58–2.62)	1.38	1.43 (0.75–2.75)
**γ-HPV 4**						
Negative	1.00	1.00	1.00	1.00	1.00	1.00
Positive	0.74	0.69 (0.31–1.54)	1.01	1.04 (0.63–1.72)	0.96	1.00 (0.64–1.57)
**μ-HPV 1**						
Negative	1.00	1.00	1.00	1.00	1.00	1.00
Positive	**0.38**	0.62 (0.23–1.65)	0.76	0.79 (0.46–1.37)	**0.65**	0.78 (0.48–1.28)
**ν-HPV 41**						
Negative	1.00	1.00	1.00	1.00	1.00	1.00
Positive	0.67	0.78 (0.25–2.47)	0.75	0.67 (0.31–1.45)	0.75	0.74 (0.38–1.44)
**β-HPV 5**						
Negative	1.00	1.00	1.00	1.00	1.00	1.00
Positive	0.67	1.01 (0.27–3.79)	0.90	1.02 (0.45–2.3)	0.87	1.02 (0.49–2.11)
**β-HPV 8**						
Negative	1.00	1.00	1.00	1.00	1.00	1.00
Positive	1.22	1.50 (0.64–3.50)	1.22	1.55 (0.88–2.76)	1.25	1.62 (0.98–2.69)
**β-HPV 12**						
Negative	1.00	1.00	1.00	1.00	1.00	1.00
Positive	1.51	1.41 (0.40–4.97)	0.80	0.60 (0.19–1.92)	1.08	0.90 (0.37–2.18)
**β-HPV 14**						
Negative	1.00	1.00	1.00	1.00	1.00	1.00
Positive	1.31	1.60 (0.38–6.72)	0.37	0.19 (0.02–1.53)	0.75	0.64 (0.21–1.94)
**β-HPV 17**						
Negative	1.00	1.00	1.00	1.00	1.00	1.00
Positive	0.77	0.80 (0.28–2.30)	1.21	1.20 (0.65–2.23)	1.12	1.12 (0.64–1.94)
**β-HPV 22**						
Negative	1.00	1.00	1.00	1.00	1.00	1.00
Positive	1.05	1.52 (0.37–6.13)	1.42	1.50 (0.61–3.68)	1.37	1.50 (0.67–3.35)
**β-HPV 23**						
Negative	1.00	1.00	1.00	1.00	1.00	1.00
Positive	0.80	1.14 (0.35–3.74)	1.40	1.27 (0.63–2.57)	1.16	1.10 (0.57–2.12)
**β-HPV 24**						
Negative	1.00	1.00	1.00	1.00	1.00	1.00
Positive	0.42	0.46 (0.05–4.14)	0.95	0.96 (0.32–2.86)	0.88	0.91 (0.34–2.44)
**β-HPV 38**						
Negative	1.00	1.00	1.00	1.00	1.00	1.00
Positive	1.33	1.82 (0.71–4.69)	0.98	0.82 (0.40–1.66)	1.04	0.96 (0.52–1.76)
**β-HPV 47**						
Negative	1.00	1.00	1.00	1.00	1.00	1.00
Positive	0.54	0.66 (0.20–2.14)	0.80	0.88 (0.44–1.79)	0.73	0.85 (0.45–1.61)
**Seropositivity to 1, 2, 3 or more HPV**						
Negative	1.00	1.00	1.00	1.00	1.00	1.
Positive to 1 HPV	0.48	0.50 (0.21–1.22)	0.82	0.82 (0.45–1.51)	0.67	0.71 (0.42–1.20)
Positive to 2 HPV	0.43	0.33 (0.08–1.30)	1.23	1.37 (0.70–2.75)	0.92	1.08 (0.56–2.03)
Positive to 3 or more HPV	0.77	1.23 (0.47–3.21)	1.08	1.12 (0.57–2.23)	0.97	1.21 (0.67–2.17)

EGLs = external genital lesions; PeIN = penile intraepithelial neoplasia; OR = odds ratios unadjusted; AOR = adjusted odds ratios

^a.^ Due to fewer cases unadjusted and adjusted models were not estimated for PeIN

^b.^ This category is also referred as ‘other EGL cases’ included different other diagnoses i.e. molluscum contagiosum, intradermal nevus, fibroepithelial polyp (skin tag), chronic balanitis, genital melanotic macule, psoriasiform dermatitis, lichenoid tissue reaction, and acute mucositis.

^c.^ This category included condyloma and suggestive of condyloma. Condyloma: A lesion with koilocytes, papillomatosis, hypergranulosis, parakeratosis and dilated blood vessels. Suggestive of condyloma: A lesion without koilocytes but with one or two of the other features associated with a condyloma. These lesions were most likely early condyloma that did not show complete histological features of a fully developed condyloma.

^d.^ Included all pathological diagnoses of EGLs

^e.^ Odds ratios were adjusted for Country of residence, age, education, circumcision status, lifetime number of female sex partners, and lifetime number of male anal sex partners.

In multivariable analyses a non-significant pattern of elevated risk was observed for any-β HPV and α-HPV type 27 seropositivity in all categories of diagnoses compared to controls. For the ‘all EGL cases’ category (AOR 1.46; 95%CI: 0.95–2.24) and AOR 1.43; 95%CI: 0.75–2.75); for ‘combined condyloma’ category (AOR 1.53; 95% CI: 0.95–2.47) and (AOR 1.24; 95% CI: 0.58–2.62); and for ‘other EGL cases’ category (AOR 1.23; 95% CI: 0.59–2.56) and (AOR 2.31; 95% CI: 0.88–6.05) respectively. No significant differences were observed when the association between grouped and type-specific cutaneous HPV seropositivity was examined with separate categories of condyloma, and suggestive of condyloma compared to controls data are available in S1 Table. Furthermore, no significant association was observed between grouped and type-specific cutaneous HPV seropositivity and condyloma when condyloma was stratified by tissue HPV 6/11 DNA positivity or antibody status to HPV 6 /11 data are available in S2 Table. Out of the 109 combined condyloma cases examined in this study, 71 (65.1%) were tissues positive for HPV 6/11 DNA, and 21 (19.3%) men with condyloma were also seropositive for HPV 6/11. No significant interaction by seropositivity to ≥1 nine vaccine type HPV was observed; therefore, the interaction terms were removed from the final models.

## Discussion

This is the first study to examine the association between cutaneous HPV seropositivity and EGLs in a prospective, multi-national cohort of men. Overall, the seroprevalence of grouped and type-specific HPV was similar across different EGL categories and controls with the most frequent types being ɤ-HPV 4, μ-HPV 1, and β-HPV 8. We did not observe an association between any-HPV, any-β HPV, and type-specific HPV seropositivity and different pathological diagnoses of EGLs [i) other EGL cases, ii) combined condyloma, and iii) all EGLs cases]. However, a non-significant pattern of elevated risk was observed for any-β HPV and α-HPV type 27 seropositivity in all categories of EGLs compared to controls. In contrast, a non-significant pattern of reduced risk was observed for μ-HPV 1 seropositivity in all categories of EGLs.

As this is the first study evaluating the role of antibodies to cutaneous HPV in the development of genital lesions located on UV-unexposed skin, no published literature exists that we can use to contextualize our findings. However, there exists a considerable amount of literature on the role of cutaneous HPV and squamous epithelial lesions of skin located on UV-exposed areas of the body that we can use to compare and contrast our findings with. The null findings in this study suggest that cutaneous HPV is not associated with EGLs on UV-unexposed skin in contrast to previous studies of other cutaneous lesions of UV-exposed skin. Reasons for these differences may include anatomic location of the lesions (i.e. UV exposed versus unexposed), immune status of the participants, and different high-risk groups of populations as previously reported [38].

It is hypothesized that cutaneous HPV infection might modify the effect of UV-induced DNA damage and apoptosis leading to accumulation of mutations and cancer initiation [39]. If UV exposure is necessary for cutaneous HPV carcinogenicity, lesions on the genital skin do not have significant UV exposure; therefore, EGLs may not be associated with exposure to cutaneous HPV infections. It is also important to mention that findings from research examining the role of cutaneous HPV in skin lesions (on UV-exposed areas of the body) are not conclusive and do not offer robust evidence for pathogenesis of these viruses. For example, different studies have reported a variety of individual types of cutaneous HPV to be associated with the SCC of the skin [5, 8, 11, 36]. Some of this variation in the type-specific associations across studies may be explained in part due to differences in the assays used, study population, age distribution, and cut-points used for seropositivity. However, it is also suggested that unlike mucosal HPV type 16 which is consistently causally associated with various forms of cancers, cutaneous HPV may act as a group of similar viruses with possible oncogenic properties [11]. Contrary to skin cancer studies [9, 11, 40] that have reported grouped cutaneous HPV association with SCC of the skin, in our study seropositivity to grouped cutaneous HPV was not associated with the risk of subsequent development of EGLs.

Cutaneous HPV DNA is abundantly found on skin surfaces and persists in hair follicles of healthy individuals [41]. This ubiquity may largely reflect viral colonization. Also, prevalence based on DNA detection varies considerably between various types of specimens collected from skin, e.g. skin swab vs. biopsy, making prevalence based on DNA detection a less precise measure. Serologic studies measuring antibodies against type-specific L1 major capsid protein of HPV provide evidence of host immune response to previous or recent infection and indicate cumulative exposure to HPV over time. Prevalence based on serology is also not dependent on the anatomic site of HPV infection. However, there are two important limitations to HPV serology studies. All infected individuals do not develop antibodies against HPV infection and antibodies may wane over time. Also, in this study we included only 14 types of cutaneous HPV, out of which 10 were from genus beta. In contrast, studies on skin cancer generally have included more cutaneous HPV types from genus beta [8, 11, 42]. This difference may partially explain the grouped cutaneous HPV associations observed in the studies on skin cancer.

In this work a non-significant pattern of reduced risk of EGLs for some HPV types was observed. Previously a few studies have also reported a protective effect for some cutaneous HPV types against skin lesions. For example, Masini et al. [43] reported a protective effect for β-HPV 15 seropositivity against SCC of the skin (OR 0.40; 95% CI: 0.20–0.90). In a recent study by Pierce-Campbell et al (*manuscript under review*), in the same *HIM Study* population, men with condyloma were significantly less likely to have beta-HPV 24 and 47 detected on the surface of the lesion than on the normal genital skin of the controls(OR: 0.09, 95% CI: 0.01–0.60 and OR: 0.20, 95% CI: 0.02–0.87, respectively).

High seroprevalence estimates in both cases and controls in the current study may suggest that cutaneous HPV are ubiquitous on skin and that cutaneous HPV might be a commensal component of the microbiological flora of the skin [44, 45]. It is also possible that cutaneous HPV might be competing against less prevalent and higher risk mucosal HPV types and some types might be involved in a protective role in the skin flora. There is a growing body of literature suggesting that non-oncogenic HPV might be stimulating or inhibiting a co-existing oncogenic HPV through interference or immune cross-reaction [46, 47]. However, all these proposed hypotheses need rigorous investigations before meaningful conclusions can be drawn. We also examined the association of grouped and type-specific cutaneous HPV seropositivity and condyloma with definite HPV etiology, categorizing condyloma by tissue DNA positivity to HPV 6/11, and antibody status to HPV 6/11, but no association was observed.

The current study is an extension of our previous work examining the role of cutaneous HPV in the development of external genital lesions in men. Biopsy-confirmed incident EGLs, prospective evaluation of the association, and the use of a previously validated [34, 42], highly sensitive assay to measure antibodies against L1 cutaneous HPV major capsid protein, are the strengths of this study. Also, important cutaneous HPV types were included in this study based on previous reports of their associations with SCC [8, 11], and recently their detection on the surface of EGLs [17]. There are also some limitations to this study. The small sample size of the PeIN (n = 6) limited our ability to further conduct subgroup and type-specific analyses for PeIN. Antibodies against cutaneous HPV are considered a marker of lifetime exposure to HPV, however, baseline serostatus in our study may have been affected by the seroconversion rate or waning of antibodies over time. Data on the serodynamics of cutaneous HPV do not exist to allow us to evaluate the effect of differential seroconversion rates and waning of antibodies over time in EGL cases and controls. Also, the anatomic site of HPV infection cannot be determined, a limitation relevant to all serological studies.

Our findings show that cutaneous HPV are not associated with the development of EGLs. Further research is need to understand the natural history and seroepidemiology of HPV infections and their role in the development of skin lesions and EGLs among men utilizing longitudinal study designs with repeated measures.

### Conclusions

Overall, exposure to cutaneous HPV was common among men; however, it appears that cutaneous HPV is not associated with the development of genital lesions in men. Further investigations are required to determine the role of cutaneous HPV in the development of skin tumors in prospective settings.

## Supporting Information

S1 Table Association between grouped and type-specific cutaneous HPV serostatus and condyloma and suggestive of condyloma compared to controls. (DOCX)

S2 Table Association between cutaneous HPV seropositivity and condyloma stratified by tissue DNA positivity to HPV 6 or 11, and serostatus to HPV 6/11. (DOCX)

## References

[1] Van Doorslaer K, Tan Q, Xirasagar S, Bandaru S, Gopalan V, Mohamoud Y, et al The Papillomavirus Episteme: a central resource for papillomavirus sequence data and analysis. Nucleic Acids Res. 2013;41(Database issue):D571–8. 10.1093/nar/gks984 23093593 PMC3531071

[2] Bernard HU, Burk RD, Chen Z, van Doorslaer K, zur Hausen H, de Villiers EM. Classification of papillomaviruses (PVs) based on 189 PV types and proposal of taxonomic amendments. Virology. 2010;401(1):70–9. 10.1016/j.virol.2010.02.002 20206957 PMC3400342

[3] Chouhy D, Bolatti EM, Perez GR, Giri AA. Analysis of the genetic diversity and phylogenetic relationships of putative human papillomavirus types. J Gen Virol. 2013;94(Pt 11):2480–8. 10.1099/vir.0.055137-0 23997181

[4] de Villiers EM, Gunst K. Characterization of seven novel human papillomavirus types isolated from cutaneous tissue, but also present in mucosal lesions. J Gen Virol. 2009;90(Pt 8):1999–2004. Epub 2009/04/24. 10.1099/vir.0.011478-0 19386784

[5] Andersson K, Waterboer T, Kirnbauer R, Slupetzky K, Iftner T, de Villiers EM, et al Seroreactivity to cutaneous human papillomaviruses among patients with nonmelanoma skin cancer or benign skin lesions. Cancer epidemiology, biomarkers & prevention: a publication of the American Association for Cancer Research, cosponsored by the American Society of Preventive Oncology. 2008;17(1):189–95. 10.1158/1055-9965.EPI-07-0405 18199724

[6] Feltkamp MC, Broer R, di Summa FM, Struijk L, van der Meijden E, Verlaan BP, et al Seroreactivity to epidermodysplasia verruciformis-related human papillomavirus types is associated with nonmelanoma skin cancer. Cancer research. 2003;63(10):2695–700. 12750299

[7] Proby CM, Harwood CA, Neale RE, Green AC, Euvrard S, Naldi L, et al A case-control study of betapapillomavirus infection and cutaneous squamous cell carcinoma in organ transplant recipients. Am J Transplant. 2011;11(7):1498–508. Epub 2011/07/02. 10.1111/j.1600-6143.2011.03589.x 21718442

[8] Iannacone MR, Gheit T, Waterboer T, Giuliano AR, Messina JL, Fenske NA, et al Case-control study of cutaneous human papillomaviruses in squamous cell carcinoma of the skin. Cancer epidemiology, biomarkers & prevention: a publication of the American Association for Cancer Research, cosponsored by the American Society of Preventive Oncology. 2012;21(8):1303–13. 10.1158/1055-9965.EPI-12-0032 PMC4543310 22707711

[9] Karagas MR, Waterboer T, Li Z, Nelson HH, Michael KM, Bavinck JN, et al Genus beta human papillomaviruses and incidence of basal cell and squamous cell carcinomas of skin: population based case-control study. Bmj. 2010;341:c2986 10.1136/bmj.c2986 20616098 PMC2900549

[10] Asgari MM, Kiviat NB, Critchlow CW, Stern JE, Argenyi ZB, Raugi GJ, et al Detection of human papillomavirus DNA in cutaneous squamous cell carcinoma among immunocompetent individuals. J Invest Dermatol. 2008;128(6):1409–17. Epub 2008/01/11. 10.1038/sj.jid.5701227 18185530 PMC3268673

[11] Waterboer T, Abeni D, Sampogna F, Rother A, Masini C, Sehr P, et al Serological association of beta and gamma human papillomaviruses with squamous cell carcinoma of the skin. The British journal of dermatology. 2008;159(2):457–9. Epub 2008/05/28. 10.1111/j.1365-2133.2008.08621.x 18503604

[12] Quint KD, Genders RE, de Koning MN, Borgogna C, Gariglio M, Bouwes Bavinck JN, et al Human Beta-papillomavirus infection and keratinocyte carcinomas. J Pathol. 2015;235(2):342–54. 10.1002/path.4425 25131163

[13] Kreuter A, Brockmeyer NH, Weissenborn SJ, Gambichler T, Stucker M, Altmeyer P, et al Penile intraepithelial neoplasia is frequent in HIV-positive men with anal dysplasia. J Invest Dermatol. 2008;128(9):2316–24. 10.1038/jid.2008.72 18385760

[14] Wieland U, Jurk S, Weissenborn S, Krieg T, Pfister H, Ritzkowsky A. Erythroplasia of queyrat: coinfection with cutaneous carcinogenic human papillomavirus type 8 and genital papillomaviruses in a carcinoma in situ. J Invest Dermatol. 2000;115(3):396–401. 10.1046/j.1523-1747.2000.00069.x 10951274

[15] Humbey O, Cairey-Remonnay S, Guerrini JS, Algros MP, Mougin C, Bittard H, et al Detection of the human papillomavirus and analysis of the TP53 polymorphism of exon 4 at codon 72 in penile squamous cell carcinomas. Eur J Cancer. 2003;39(5):684–90. 12628849 10.1016/s0959-8049(02)00835-3

[16] Heideman DA, Waterboer T, Pawlita M, Delis-van Diemen P, Nindl I, Leijte JA, et al Human papillomavirus-16 is the predominant type etiologically involved in penile squamous cell carcinoma. J Clin Oncol. 2007;25(29):4550–6. 10.1200/JCO.2007.12.3182 17925550

[17] Pierce Campbell CM, Messina JL, Stoler MH, Jukic DM, Tommasino M, Gheit T, et al Cutaneous human papillomavirus types detected on the surface of male external genital lesions: a case series within the HPV Infection in Men Study. Journal of clinical virology: the official publication of the Pan American Society for Clinical Virology. 2013;58(4):652–9. Epub 2013/11/12. 24210970 10.1016/j.jcv.2013.10.011 PMC3866698

[18] Harwood CA, Spink PJ, Surentheran T, Leigh IM, de Villiers EM, McGregor JM, et al Degenerate and nested PCR: a highly sensitive and specific method for detection of human papillomavirus infection in cutaneous warts. J Clin Microbiol. 1999;37(11):3545–55. 10523550 10.1128/jcm.37.11.3545-3555.1999 PMC85688

[19] IARC. Human papillomaviruses: Iarc Working Group on the Evaluation of Carcinogenic Risks to Humans. 2007;90:1–636. PMC4781057 18354839

[20] Hernandez BY, Barnholtz-Sloan J, German RR, Giuliano A, Goodman MT, King JB, et al Burden of invasive squamous cell carcinoma of the penis in the United States, 1998–2003. Cancer. 2008;113(10 Suppl):2883–91. 10.1002/cncr.23743 18980292 PMC2693711

[21] Misra S, Chaturvedi A, Misra NC. Penile carcinoma: a challenge for the developing world. The Lancet Oncology. 2004;5(4):240–7. 10.1016/S1470-2045(04)01427-5 15050955

[22] Baldur-Felskov B, Hannibal CG, Munk C, Kjaer SK. Increased incidence of penile cancer and high-grade penile intraepithelial neoplasia in Denmark 1978–2008: a nationwide population-based study. Cancer causes & control: CCC. 2012;23(2):273–80. 22101453 10.1007/s10552-011-9876-7

[23] Graafland NM, Verhoeven RH, Coebergh JWW, Horenblas S. Incidence trends and survival of penile squamous cell carcinoma in the Netherlands. International Journal of Cancer. 2011;128(2):426–32. 10.1002/ijc.25355 20340128

[24] Arya M, Kalsi J, Kelly J, Muneer A. Malignant and premalignant lesions of the penis. Bmj. 2013;346:f1149 10.1136/bmj.f1149 23468288

[25] Insinga RP, Dasbach EJ, Myers ER. The health and economic burden of genital warts in a set of private health plans in the United States. Clin Infect Dis. 2003;36(11):1397–403. 10.1086/375074 12766834

[26] Blomberg M, Friis S, Munk C, Bautz A, Kjaer SK. Genital warts and risk of cancer: a Danish study of nearly 50 000 patients with genital warts. The Journal of infectious diseases. 2012;205(10):1544–53. 10.1093/infdis/jis228 22427679

[27] Cubilla AL, Velazquez EF, Young RH. Epithelial lesions associated with invasive penile squamous cell carcinoma: a pathologic study of 288 cases. International journal of surgical pathology. 2004;12(4):351–64. 15494861 10.1177/106689690401200408

[28] Giuliano AR, Lazcano-Ponce E, Villa LL, Flores R, Salmeron J, Lee JH, et al The human papillomavirus infection in men study: human papillomavirus prevalence and type distribution among men residing in Brazil, Mexico, and the United States. Cancer epidemiology, biomarkers & prevention: a publication of the American Association for Cancer Research, cosponsored by the American Society of Preventive Oncology. 2008;17(8):2036–43. 10.1158/1055-9965.EPI-08-0151 PMC3471778 18708396

[29] Giuliano AR, Lee JH, Fulp W, Villa LL, Lazcano E, Papenfuss MR, et al Incidence and clearance of genital human papillomavirus infection in men (HIM): a cohort study. Lancet. 2011;377(9769):932–40. 10.1016/S0140-6736(10)62342-2 21367446 PMC3231998

[30] Ingles DJ, Pierce Campbell CM, Messina JA, Stoler MH, Lin HY, Fulp WJ, et al Human papillomavirus virus (HPV) genotype- and age-specific analyses of external genital lesions among men in the HPV Infection in Men (HIM) Study. The Journal of infectious diseases. 2015;211(7):1060–7. 10.1093/infdis/jiu587 25344518 PMC4432433

[31] Sudenga SL, Ingles DJ, Pierce Campbell CM, Lin HY, Fulp WJ, Messina JL, et al Genital Human Papillomavirus Infection Progression to External Genital Lesions: The HIM Study. Eur Urol. 2015. 10.1016/j.eururo.2015.05.032 PMC4670812 26051441

[32] Anic GM, Messina JL, Stoler MH, Rollison DE, Stockwell H, Villa LL, et al Concordance of human papillomavirus types detected on the surface and in the tissue of genital lesions in men. J Med Virol. 2013;85(9):1561–6. 10.1002/jmv.23635 23852680 PMC3879682

[33] Sehr P, Zumbach K, Pawlita M. A generic capture ELISA for recombinant proteins fused to glutathione S-transferase: validation for HPV serology. J Immunol Methods. 2001;253(1–2):153–62. 11384677 10.1016/s0022-1759(01)00376-3

[34] Sehr P, Muller M, Hopfl R, Widschwendter A, Pawlita M. HPV antibody detection by ELISA with capsid protein L1 fused to glutathione S-transferase. Journal of virological methods. 2002;106(1):61–70. 12367730 10.1016/s0166-0934(02)00134-9

[35] Waterboer T, Sehr P, Michael KM, Franceschi S, Nieland JD, Joos TO, et al Multiplex human papillomavirus serology based on in situ-purified glutathione s-transferase fusion proteins. Clin Chem. 2005;51(10):1845–53. 10.1373/clinchem.2005.052381 16099939

[36] Casabonne D, Michael KM, Waterboer T, Pawlita M, Forslund O, Burk RD, et al A prospective pilot study of antibodies against human papillomaviruses and cutaneous squamous cell carcinoma nested in the Oxford component of the European Prospective Investigation into Cancer and Nutrition. Int J Cancer. 2007;121(8):1862–8. 10.1002/ijc.22885 17565742

[37] Michael KM, Waterboer T, Sehr P, Rother A, Reidel U, Boeing H, et al Seroprevalence of 34 human papillomavirus types in the German general population. PLoS pathogens. 2008;4(6):e1000091 10.1371/journal.ppat.1000091 18566657 PMC2408730

[38] Majewski S, Jablonska S. Skin autografts in epidermodysplasia verruciformis: human papillomavirus-associated cutaneous changes need over 20 years for malignant conversion. Cancer Res. 1997;57(19):4214–6. 9331078

[39] Aldabagh B, Angeles JG, Cardones AR, Arron ST. Cutaneous squamous cell carcinoma and human papillomavirus: is there an association? Dermatologic surgery: official publication for American Society for Dermatologic Surgery [et al]. 2013;39(1 Pt 1):1–23. Epub 2012/08/30. 10.1111/j.1524-4725.2012.02558.x PMC3521067 22928516

[40] Karagas MR, Nelson HH, Sehr P, Waterboer T, Stukel TA, Andrew A, et al Human papillomavirus infection and incidence of squamous cell and basal cell carcinomas of the skin. Journal of the National Cancer Institute. 2006;98(6):389–95. 10.1093/jnci/djj092 16537831

[41] Plasmeijer EI, Struijk L, Bouwes Bavinck JN, Feltkamp MC. Epidemiology of cutaneous human papillomavirus infections. Cancer Treat Res. 2009;146:143–57. 10.1007/978-0-387-78574-5_13 19415200

[42] Waterboer T, Neale R, Michael KM, Sehr P, de Koning MN, Weissenborn SJ, et al Antibody responses to 26 skin human papillomavirus types in the Netherlands, Italy and Australia. J Gen Virol. 2009;90(Pt 8):1986–98. Epub 2009/04/24. 10.1099/vir.0.010637-0 19386782

[43] Masini C, Fuchs PG, Gabrielli F, Stark S, Sera F, Ploner M, et al Evidence for the association of human papillomavirus infection and cutaneous squamous cell carcinoma in immunocompetent individuals. Arch Dermatol. 2003;139(7):890–4. 10.1001/archderm.139.7.890 12873884

[44] Hannigan GD, Grice EA. Microbial ecology of the skin in the era of metagenomics and molecular microbiology. Cold Spring Harb Perspect Med. 2013;3(12):a015362 10.1101/cshperspect.a015362 24296350 PMC3839604

[45] Antonsson A, Forslund O, Ekberg H, Sterner G, Hansson BG. The ubiquity and impressive genomic diversity of human skin papillomaviruses suggest a commensalic nature of these viruses. J Virol. 2000;74(24):11636–41. 11090162 10.1128/jvi.74.24.11636-11641.2000 PMC112445

[46] Accardi R, Gheit T. Cutaneous HPV and skin cancer. Presse medicale (Paris, France: 1983). 2014;43(12P2):e435–e43. Epub 2014/12/03. 10.1016/j.lpm.2014.08.008 25451638

[47] Mejlhede N, Pedersen BV, Frisch M, Fomsgaard A. Multiple human papilloma virus types in cervical infections: competition or synergy? APMIS. 2010;118(5):346–52. 10.1111/j.1600-0463.2010.02602.x 20477809

