# Supplementary material for: Seroprevalence of Cutaneous Human Papillomaviruses and the Risk of External Genital Lesions in Men: A Nested Case-Control Study
Source: PLoS One. 2016 Nov 28;11(11):e0167174. doi: 10.1371/journal.pone.0167174 (PMC5125700; doi:10.1371/journal.pone.0167174)
Supplement: S1 Table — (DOCX) [file pone.0167174.s001.docx]

**S1 Table. Association between grouped and type-specific cutaneous HPV serostatus and condyloma and suggestive of condyloma compared to controls**^a^

| **HPV type** | **Condyloma^b^ (N=62) Controls (N=352)** | **Suggestive of Condyloma**^c^ **(N=47) Controls (N=352)** |
| --- | --- | --- |
|  | **OR 95% CI** | **OR 95% CI** |
| **Any-HPV** |  |  |
| Negative | 1.00 | 1.00 |
| Positive | 1.47 ( 0.77 - 2.84) | 0.63 ( 0.33 - 1.19) |
| **Any β HPV** |  |  |
| Negative | 1.00 | 1.00 |
| Positive | 1.71 ( 1.00 - 2.95) | 1.09 ( 0.59 - 2.03) |
| **α-HPV 27** |  |  |
| Negative | 1.00 | 1.00 |
| Positive | 1.39 ( 0.61 - 3.15) | 1.11 ( 0.41 – 3.00) |
| **γ-HPV 4** |  |  |
| Negative | 1.00 | 1.00 |
| Positive | 1.23 ( 0.70 - 2.16) | 0.76 ( 0.38 - 1.53) |
| **µ-HPV 1** |  |  |
| Negative | 1.00 | 1.00 |
| Positive | 0.97 ( 0.54 - 1.75) | 0.52 ( 0.24 - 1.11) |
| **η-HPV 41** |  |  |
| Negative | 1.00 | 1.00 |
| Positive | 0.79 ( 0.32 - 1.95) | 0.69 ( 0.23 - 2.01) |
| **β-HPV 5** |  |  |
| Negative | 1.00 | 1.00 |
| Positive | 0.88 ( 0.33 - 2.35) | 0.93 ( 0.31 - 2.76) |
| **β-HPV 8** |  |  |
| Negative | 1.00 | 1.00 |
| Positive | 0.98 ( 0.50 - 1.95) | 1.57 ( 0.79 - 3.13) |
| **β-HPV 12** |  |  |
| Negative | 1.00 | 1.00 |
| Positive | 0.84 ( 0.24 - 2.93) | 0.74 ( 0.17 - 3.26) |
| **β-HPV 14** |  |  |
| Negative | 1.00 | 1.00 |
| Positive | 0.66 ( 0.15 - 2.92) | 0.00 ( NE) |
| **β-HPV 17** |  |  |
| Negative | 1.00 | 1.00 |
| Positive | 1.16 ( 0.57 - 2.37) | 1.28 ( 0.59 - 2.79) |
| **β-HPV 22** |  |  |
| Negative | 1.00 | 1.00 |
| Positive | 1.09 ( 0.36 - 3.28) | 1.88 ( 0.67 - 5.24) |
| **β-HPV 23** |  |  |
| Negative | 1.00 | 1.00 |
| Positive | 1.12 ( 0.47 - 2.64) | 1.8 ( 0.78 - 4.15) |
| **β-HPV 24** |  |  |
| Negative | 1.00 | 1.00 |
| Positive | 1.00 ( 0.28 - 3.53) | 0.88 ( 0.20 - 3.92) |
| **β-HPV 38** |  |  |
| Negative | 1.00 | 1.00 |
| Positive | 0.85 ( 0.36 - 1.97) | 1.16 ( 0.49 - 2.75) |
| **β-HPV 47** |  |  |
| Negative | 1.00 | 1.00 |
| Positive | 1.13 ( 0.54 - 2.38) | 0.4 ( 0.12 - 1.34) |
| **Seroactivity to 1, 2, 3 or more HPV** |  |  |
| Negative | 1.00 | 1.00 |
| Positive to 1 HPV | 1.40 ( 0.68 - 2.91) | 0.40 ( 0.17 - 0.92) |
| Positive to 2 HPV | 1.87 ( 0.82 - 4.26) | 0.77 ( 0.32 - 1.89) |
| Positive to 3 or more HPV | 1.29 ( 0.55 - 2.99) | 0.93 ( 0.42 - 2.05) |
| OR = Odds Ratios unadjusted ; 95%CI = 95% Confidence Intervals  a. Controls included men who did not develop any EGL in the entire follow up period.  b. Condyloma: A lesion with koilocytes, papillomatosis, hypergranulosis, parakeratosis and dilated blood vessels.  c. Suggestive of condyloma: A lesion without koilocytes but with one or two of the other features associated with a condyloma. These lesions were categorized as squamous keratosis or benign squamous papilloma. They are most likely early condyloma that did not show complete histological features of a fully developed condyloma. | | |
