# Supplementary material for: Seroprevalence of Cutaneous Human Papillomaviruses and the Risk of External Genital Lesions in Men: A Nested Case-Control Study
Source: PLoS One. 2016 Nov 28;11(11):e0167174. doi: 10.1371/journal.pone.0167174 (PMC5125700; doi:10.1371/journal.pone.0167174)
Supplement: S2 Table — (DOCX) [file pone.0167174.s002.docx]

**S2 Table. Association between cutaneous HPV seropositivity and condyloma stratified by tissue DNA positivity to HPV 6 or 11, and serostatus to HPV6/11**

|  | **Condyloma Stratified by Tissues DNA Positivity to HPV 6/11**^a^ | **Condyloma Stratified by Serostatus to HPV 6/11**^b^ |
| --- | --- | --- |
| **HPV type** | **OR (95%CI)**^c^ | **OR (95%CI)**^d^ |
| **Any-HPV** |  |  |
| Seronegative | 1.00 | 1.00 |
| Seropositive | 0.69 ( 0.28 - 1.70) | 1.34 ( 0.45 - 4.05) |
| **Any-β** |  |  |
| Seronegative | 1.00 | 1.00 |
| Seropositive | 0.70 ( 0.32 - 1.54) | 2.14 ( 0.81 - 5.68) |
| **α-HPV 27** |  |  |
| Seronegative | 1.00 | 1.00 |
| Seropositive | 1.23 ( 0.35 - 4.31) | 1.30 ( 0.32 - 5.21) |
| **γ-HPV 4** |  |  |
| Seronegative | 1.00 | 1.00 |
| Seropositive | 0.67 ( 0.29 - 1.55) | 0.64 ( 0.21 - 1.91) |
| **µ-HPV1** |  |  |
| Seronegative | 1.00 | 1.00 |
| Seropositive | 0.95 ( 0.39 - 2.33) | 0.88 ( 0.29 - 2.68) |
| **ν-HPV 41** |  |  |
| Seronegative | 1.00 | 1.00 |
| Seropositive | 0.78 ( 0.21 - 2.97) | 0.44 ( 0.05 - 3.67) |
| **β-HPV 5** |  |  |
| Seronegative | 1.00 | 1.00 |
| Seropositive | 1.08 ( 0.25 - 4.57) | 2.28 ( 0.52 - 9.97) |
| **β-HPV 8** |  |  |
| Seronegative | 1.00 | 1.00 |
| Seropositive | 0.75 ( 0.30 - 1.88) | 1.06 ( 0.35 - 3.26) |
| **β-HPV 12** |  |  |
| Seronegative | 1.00 | 1.00 |
| Seropositive | 0.12 ( 0.01 - 1.13) | 1.05 ( 0.11 - 9.91) |
| **β-HPV 14** |  |  |
| Seronegative | 1.00 | 1.00 |
| Seronegative | 0.53 ( 0.03 - 8.69) | 0.0 (NE) |
| **β-HPV 17** |  |  |
| Seronegative | 1.00 | 1.00 |
| Seropositive | 0.99 ( 0.36 - 2.74) | 1.52 ( 0.48 - 4.79) |
| **β-HPV 22** |  |  |
| Seronegative | 1.00 | 1.00 |
| Seropositive | 0.64 ( 0.16 - 2.56) | 2.28 ( 0.52 - 9.97) |
| **β-HPV 23** |  |  |
| Seronegative | 1.00 | 1.00 |
| Seropositive | 0.77 ( 0.25 - 2.37) | 1.65 ( 0.47 - 5.80) |
| **β-HPV 24** |  |  |
| Seronegative | 1.00 | 1.00 |
| Seropositive | 0.12 ( 0.01 - 1.13) | 2.98 ( 0.47 - 19.1) |
| **β-HPV 38** |  |  |
| Seronegative | 1.00 | 1.00 |
| Seropositive | 0.17 ( 0.05 - 0.58) | 1.84 ( 0.51 - 6.55) |
| **β-HPV 47** |  |  |
| Seronegative | 1.00 | 1.00 |
| Seropositive | 1.23 ( 0.35 - 4.31) | 1.63 ( 0.36 - 15.70) |
| **Seropositivity to 1, 2, 3 or more types** |  |  |
| Seronegative | 1.00 | 1.00 |
| Seropositive to 1 types | 1.46 ( 0.47 - 4.58) | 1.46 ( 0.41 - 5.20) |
| Seropositive to 2 types | 0.41 ( 0.13 - 1.28) | 0.82 ( 0.17 - 3.86) |
| Seropositive to 3 or more types | 0.48 ( 0.16 - 1.48) | 1.73 ( 0.46 - 6.56) |
| OR = Odds Ratios unadjusted ; CI = Confidence Intervals  a. Both condyloma and suggestive condyloma were assessed for tissue HPV 6/11 DNA. Condyloma was defined as a lesion with koilocytes, papillomatosis, hypergranulosis, parakeratosis and dilated blood vessels. Suggestive of condyloma was defined a lesion without koilocytes but with one or two of the other features associated with a condyloma. These lesions were categorized as squamous keratosis or benign squamous papilloma. They are most likely early condyloma that did not show complete histological features of a fully developed condyloma.  b. Both condyloma and suggestive condyloma were assessed for serostatus to HPV 6/11. Serostatus_HPV6/11 variable was created if a man with condyloma/suggestive condyloma was seropositive for HPV 6/11 then the serostatus_HPV6/11 variables was 1 else 0.  c. The reference group was condyloma negative for HPV 6/11 DNA.  d. The reference group was condyloma with serostatus negative to HPV 6/11. | | |
